# Subcortical nuclei volumes are associated with cognition in children post-convulsive status epilepticus: Results at nine years follow-up

**DOI:** 10.1016/j.yebeh.2020.107119

**Published:** 2020-09

**Authors:** Kyle H. Bennett, Suresh S. Pujar, Marina M. Martinos, Christopher A. Clark, Michael Yoong, Rod C. Scott, Richard F.M. Chin

**Affiliations:** a Muir Maxwell Epilepsy Centre, Child Life and Health, University of Edinburgh, UK; b Neurosciences Unit, Institute of Child Health, University College London, London, UK; c Cognitive Neuroscience and Neuropsychiatry Unit, Institute of Child Health, University College London, London, UK; d College of Medicine and Veterinary Medicine, University of Edinburgh, Edinburgh, UK; e Department of Paediatric Neurosciences, Royal Hospital for Sick Children, Edinburgh, UK; f Department of Neurological Sciences, University of Vermont, Burlington, VT, USA

**Keywords:** CSE, convulsive status epilepticus, FSIQ, full-scale IQ, GMS, global memory scores, PFS, prolonged febrile seizures, HC, healthy controls, SCV, total subcortical volume, CI, cognitive impairment, ICV, intracranial volume, STEPSOUT, Status Epilepticus Outcomes Study, NLSTEPSS, North London Convulsive Status Epilepticus in Childhood Surveillance Study, IMD, Index of Multiple Deprivation 2004, GM, gray matter, PIQ, performance IQ, VIQ, verbal IQ, WASI, Wechsler Abbreviated Scales of Intelligence, CMS, Children's Memory Scale, Volumetric MRI, Status epilepticus, Pediatric, Cognition, Memory, Subcortical nuclei

## Abstract

**Purpose:**

The purpose of the present study was to investigate the relationship between subcortical nuclei volume and cognition in children with post-convulsive status epilepticus (CSE).

**Methods:**

Structural T1-weighted magnetic resonance imaging (MRI) scans (Siemens Avanto, 1.5 T) and neuropsychological assessments (full-scale intelligence quotient (FSIQ) and Global Memory Scores (GMS)) were collected from subjects at a mean 8.5 years post-CSE (prolonged febrile seizures (PFS), n = 30; symptomatic/known, n = 28; and other, n = 12) and from age- and sex-matched healthy controls (HC). Subjects with CSE were stratified into those with lower cognitive ability (LCA) (CSE +, n = 22) and those without (CSE −, n = 48). Quantitative volumetric analysis using Functional MRI of the Brain Software Library (FSL) (Analysis Group, FMRIB, Oxford) provided segmented MRI brain volumes. Univariate analysis of covariance (ANCOVA) was performed to compare subcortical nuclei volumes across subgroups. Multivariable linear regression was performed for each subcortical structure and for total subcortical volume (SCV) to identify significant predictors of LCA (FSIQ < 85) while adjusting for etiology, age, socioeconomic status, sex, CSE duration, and intracranial volume (ICV); Bonferroni correction was applied for the analysis of individual subcortical nuclei.

**Results:**

Seventy subjects (11.8 ± 3.4 standard deviation (SD) years; 34 males) and 72 controls (12.1 ± 3.0SD years; 29 males) underwent analysis. Significantly smaller volumes of the left thalamus, left caudate, right caudate, and SCV were found in subjects with CSE + compared with HC, after adjustment for intracranial, gray matter (GM), or cortical/cerebellar volume. When compared with subjects with CSE −, subjects with CSE + also had smaller volumes of the left thalamus, left pallidum, right pallidum, and SCV. Individual subcortical nuclei were not associated, but SCV was associated with FSIQ (p = 0.005) and GMS (p = 0.014). Intracranial volume and etiology were similarly predictive.

**Conclusions:**

Nine years post-CSE, SCV is significantly lower in children who have LCA compared with those that do not. However, in this cohort, we are unable to determine whether the relationship is independent of ICV or etiology. Future, larger scale studies may help tease this out.

## 1 Introduction

### 1.1 Background

Convulsive status epilepticus (CSE) is the most common pediatric neurological emergency and is associated with lower cognitive ability (LCA) [1,2]. Lower cognitive ability accounts for a significant proportion of the lower quality of life reported in this population [3]. The degree of LCA is largely dependent on etiology of CSE: subjects presenting with prolonged febrile seizures (PFS) perform significantly better than those with non-PFS upon formal psychometric cognitive testing [4]. The mechanisms behind LCA are uncertain, although there is evidence that CSE may result in neuronal injury and disruption of neuronal networks [5].

Cognition is usually considered a reflection of cortical function, with studies reporting LCA in those with reduced cortical volume [6]. However, there is also evidence that subcortical structures are linked to cognition [[7], [8], [9], [10], [11]]. The role of subcortical structures in cognition has been explored within several studies of patients with neurological and/or psychiatric disease; in general, those with LCA are reported to have reduced volumes of the subcortical nuclei [[12], [13], [14], [15]]. Lower cognitive ability in various epilepsies has been associated with lower volumes of subcortical nuclei, particularly the thalamus [11,16], suggesting a cognitive role in patients with epilepsy. Other studies have found that smaller volumes of not only the thalami but also the putamina and caudate nuclei are present in patients with both febrile and nonfebrile seizures [17,18]. To the authors' knowledge, the volumes of the four subcortical structures have not yet been studied in the context of CSE and the relationship with LCA. Having recently shown in a 9-year follow-up of a unique childhood CSE cohort that children with CSE who undertook formal testing (n = 94) were shown to have significantly lower full-scale intelligence quotient (FSIQ) and Global Memory Scores (GMS) from controls, we aimed in the current study to examine the relationship between subcortical volumes and FSIQ and GMS in children who had CSE [19]. Such data may provide insight into the role of these structures in cognition and aid our understanding of the pathophysiology of LCA in CSE. Furthermore, the identification of structural abnormalities in the subcortical structures may allow for recognition of those susceptible to subsequent LCA, thereby facilitating the prioritization of earlier neuropsychological interventions.

### 1.2 Objectives

We hypothesized that subcortical nuclei volumes would correlate with FSIQ and GMS scores in children post-CSE. Therefore, we aimed to establish (1) if subcortical volumetric differences exist among healthy controls (HC), subjects with CSE with LCA (CSE +) and subjects with CSE without LCA (CSE −) and (2) to determine if there was an association between subcortical nuclei volumes and FSIQ/GMS scores at long-term follow-up post-CSE.

## 2 Materials and methods

### 2.1 Participants

Data of the present study were collected from the cohort of the Status Epilepticus Outcomes Study (STEPSOUT) [20,21], which followed up children within 10 years after CSE. These participants were originally recruited from the North London Convulsive Status Epilepticus in Childhood Surveillance Study (NLSTEPSS), the first epidemiological study focused on childhood CSE in which there are detailed prospectively collected clinical and sociodemographic data available [22]. Details on recruitment, magnetic resonance imaging (MRI) assessments and detailed neuropsychology assessments for participants in the follow-up study, STEPSOUT, have been reported elsewhere [20,22]. In short, at a mean follow-up of 9 years, subjects with CSE who were not lost to follow-up were invited for MRI on an Avanto 1.5 Tesla whole-body MRI scanner (Siemens, Erlangen, Germany) and neuropsychological assessments by an experienced psychologist (MM) at UCL Great Ormond Street Institute of Child Health (ICH) in London, United Kingdom. On attendance for assessment, participants and their parents were interviewed using standardized proformas to obtain clinical details [23]. Index of Multiple Deprivation (IMD) 2004 based on participant residential postal code was used as a measure of socioeconomic status [24].

The definitions for CSE etiologies have been given elsewhere but are combined in the current study to increase power in intergroup comparisons [4,25]. Participants were categorized based on their initial etiology [26] as follows: (1) PFS, (2) symptomatic/known (acute and remote symptomatic), and (3) other (idiopathic, cryptogenic, and unclassified etiology). This categorization is a modification of the International League Against Epilepsy (ILAE) classification of status epilepticus [5] in which we have included PFS as it is an etiology unique to childhood [25]. A convenience sample of age- and sex-matched healthy children without known neurological or developmental problems were recruited as controls (HC group); these participants were siblings of study subjects, personal contacts to authors of STEPSOUT, relatives of employees at the research center, or anonymized controls from previous studies who underwent neuropsychological assessments and MRI using the same protocol [27]. Controls were not IQ-matched with study subjects.

Potential study subjects and/or parents were provided with written information about the study. Informed written consent was obtained prior to their enrollment. The STEPSOUT was approved by the University College London Institute of Child Health/Great Ormond Street Hospital Research Ethics Committee (ref: 07NR01). All data were anonymized, and no further data were collected for the purposes of the present study.

### 2.2 Neuroimaging measures

**Fig. 1:**
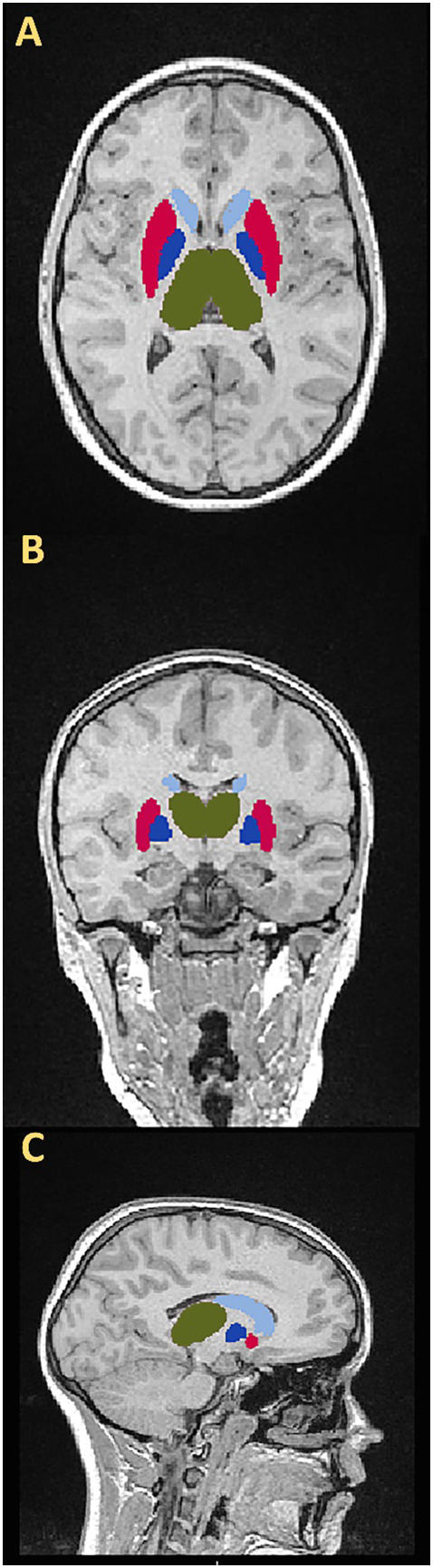
Segmented subcortical nuclei volumes of a control participant overlaid on the respective head MRI. Three planes are represented: A = axial, B = coronal, C = sagittal. Subcortical nuclei are color coded: dark green = thalami, light blue = caudate nuclei, red = putamina, dark blue = pallidi.

All MRI scan sequences were reviewed by an experienced pediatric neuroradiologist who classified scans as being either normal or showing minor/major abnormalities according to predefined criteria established for a separate CSE cohort studied by our group [28]. Subjects were excluded from the current study when major structural MRI abnormalities (including hemorrhages, atrophy, large infarcts, and hydrocephalus) precluded reliable subcortical volume measurements. In the current study, T1-weighted volumetric images were converted into Neuroimaging Informatics Technology Initiative (NIfTI-1) format and reoriented to match standard template images of FSLView (Analysis Group, Functional MRI of the Brain (FMRIB), Oxford, U.K.). The Brain Extraction Tool (BET) was utilized to remove nonbrain tissue from scans [29]. Volumes of subcortical nuclei (thalami, caudate nuclei, putamina, and pallidi) were segmented from resultant brain images using FMRIB's Integrated Registration and Segmentation Tool (FIRST) [30] (Fig. 1). Gray matter (GM), white matter, and cerebrospinal fluid (CSF) were segmented from brain images using FMRIB's Automated Segmentation Tool (FAST) and used to calculate intracranial volume (ICV) [31]. Segmented scans were manually reviewed by two of the authors (KHB, MY) by overlaying segmented subcortical nuclei with MRI head scans. Scans that precluded segmentation due to major anatomical abnormalities were discussed and excluded from analysis by consensus where manual segmentation was deemed unmanageable. KHB and MY were blinded to whether MRI data related to subjects or controls.

### 2.3 Neuropsychological evaluation

Full-scale intelligence quotient, verbal intelligence quotient (VIQ), and performance intelligence quotient (PIQ) were determined using Wechsler Abbreviated Scales of Intelligence (WASI), and GMS were assessed using the Children's Memory Scale (CMS) [19]. Both tests have a normative mean of 100 and a standard deviation (SD) of 15. In the current study, we restricted analyses on IQ to FSIQ rather than including its constituents since no discrepancies between VIQ and PIQ were observed in our sample. Lower cognitive ability was defined in the current study as FSIQ scores < 85; subjects with CSE were stratified into those with LCA (CSE +) and those without LCA (CSE −). The outcomes of interest as markers of cognition were FSIQ and GMS scores.

### 2.4 Statistical analysis

Statistical analysis was carried out using SPSS V23.0.0.0 (Armonk, New York, U.S.A., IBM Corp.). Type I error was set at α = 0.05.

To assess if there were intergroup difference in sociodemographic or clinical characteristics, we carried out comparisons of study participants according to (1) CSE, subgroups with CSE (PFS, symptomatic/known, other), and HC; (2) groups with CSE +, CSE −, and HC. Outliers were included in these analyses.

Pearson χ^2^ tests were performed for all categorical variables. Where less than 80% of cell counts were greater than 5, Fisher's exacts tests were performed instead.

For assessment of continuous variables between CSE etiology subgroups and HC, univariate one-way analysis of variance (ANOVA) tests were performed where data were normally distributed (Shapiro–Wilk p > 0.05) — time until follow-up, IMD; otherwise, Kruskall–Wallis H testing with Bonferroni post hoc testing was applied (all Levene's tests for equality of variances were p > 0.05).

For assessment of continuous variables between CSE +, CSE −, and HC, Kruskall–Wallis H testing with Bonferroni and Games–Howell post hoc testing were performed for age at follow-up as data were not normally distributed (Shapiro–Wilk p < 0.05). For all other continuous variables, independent t tests were performed for normally distributed data, and Mann–Whitney U tests were performed for all non-normally distributed data. Levene's test for equality of variances was used to determine p values for independent t tests.

To investigate differences in subcortical nuclei volumes between groups with CSE +, CSE −, and HC, univariate one-way analysis of covariance (ANCOVA) analyses were performed. We adjusted for potentially confounding variables: age at follow-up, sex, ICV, GM volume, and cortical/cerebellar volumes (GM minus total subcortical volume (SCV)). Bonferroni correction was applied to the p values for ANCOVA of individual subcortical volumes. Mean differences of subcortical volumes between groups were calculated with corresponding p values.

To investigate whether there was a group effect of CSE vs HC in factors associated with FSIQ and GMS in CSE, we carried out regression analyses including potential predictor variables for which there were data available for both groups, as well as group with CSE vs HC group as a potential predictor variable. Thus, the variables examined were CSE vs HC, age at follow-up, sex, ICV, and subcortical volumes. Etiology and IMD (socioeconomic status) were not available for controls.

Since we identified that there was a group effect from the analysis, we subsequently did further regression analyses restricted to the group with CSE only to identify characteristics of the patients with CSE, including subcortical volumes that were associated with FSIQ and GMS. Any participants with CSE with missing FSIQ/GMS data were excluded from these analyses. Analyses were initially performed for each individual subcortical nucleus (thalami, putamina, pallidi, and caudate nuclei) adjusting for age at follow-up, sex, IMD (socioeconomic status) scores, duration of CSE, ICV, and etiology. From our previous work, symptomatic/known etiology was significantly associated with outcomes. Therefore, we dichotomized etiology according to symptomatic/known vs all other types in the modeling. Duration of CSE, despite previously being shown to not be associated with long-term changes, represents a modifiable factor by means of pharmacological intervention, so it was included in models [4,32]. The other included variables have been reported in the literature as significantly associated with cognition. Bonferroni correction was applied to resultant p-values of linear regressions for individual subcortical volumes. As there was significant collinearity between subcortical nuclei volumes (Supporting Information 1), the SCV, the combined volume of all subcortical structures, was used on its own in a further set of linear regression analyses as a predictor variable.

There was, however, significant collinearity between SCV and ICV (Pearson's coefficient = 0.797, p < 0.001). Each was also collinear with the symptomatic/known vs non-symptomatic/known etiology (Pearson's coefficient = − 0.310, p = 0.005; Pearson's coefficient = − 0.221, p = 0.033, respectively), which precluded inclusion of more than one of each in the multiple regression model (Supporting Information 1). To determine which of the three remained preferentially significant, we applied a stepwise regression approach in the final modeling.

## 3 Results

### 3.1 Participants

**Fig. 2:**
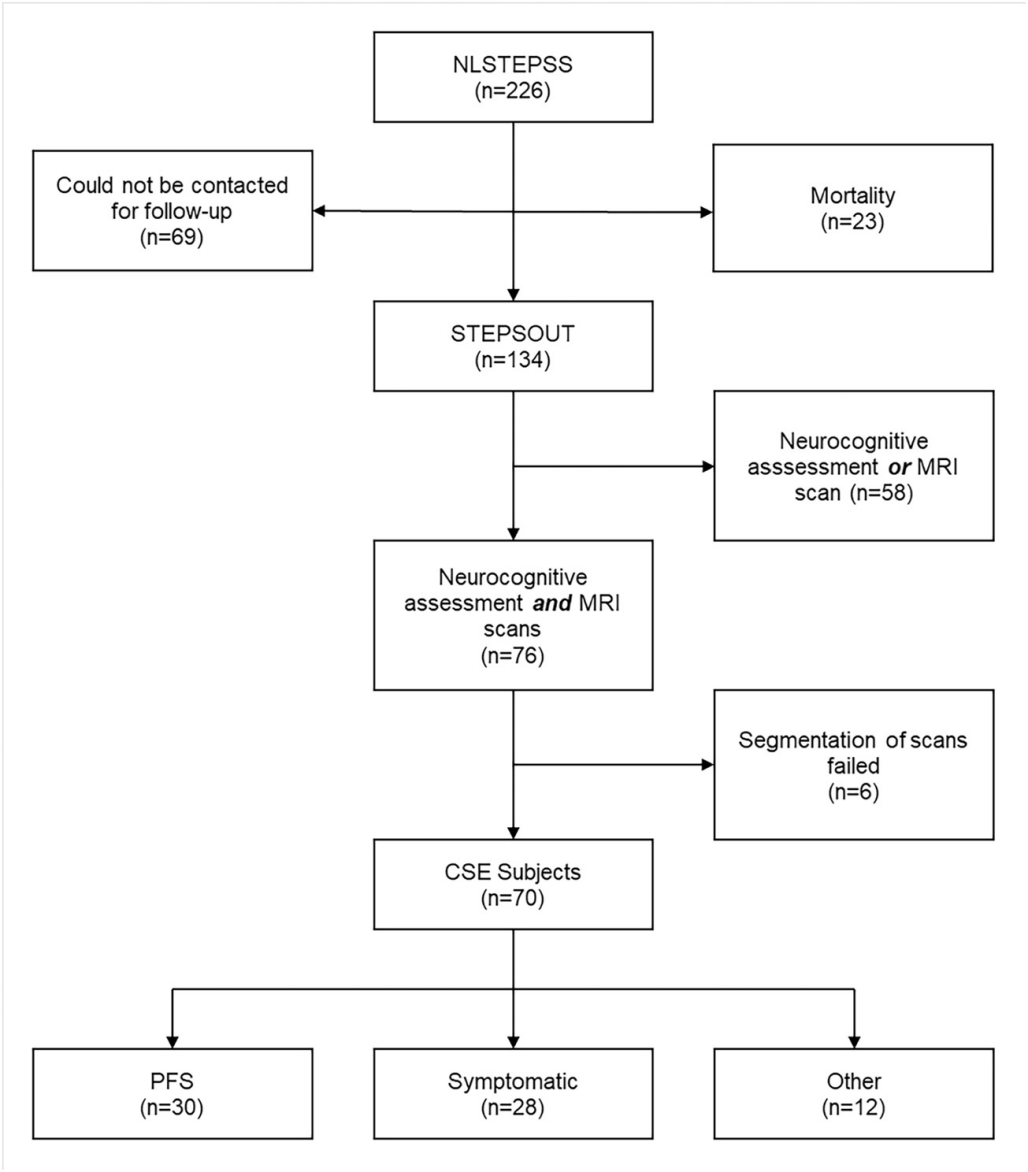
Selection of patients with CSE from the inception cohort, NLSTEPSS, and the follow-up study, STEPSOUT. NLSTEPSS = North London Convulsive Status Epilepticus in Childhood Surveillance Study; STEPSOUT = Status Epilepticus Outcomes Study; CSE = convulsive status epilepticus.

Of the 134 participants included in STEPSOUT, 76 who had childhood CSE underwent both neurocognitive assessment and MRI scans (Fig. 2). Subjects in whom segmentation of their scans was not possible because of abnormal neuroanatomy were excluded (n = 6) (Supporting Information 2 and 3). A total of 70 subjects with CSE (52% of STEPSOUT) and 72 HC were included in the current study. The group with CSE comprised the following etiology subgroups: PFS (n = 30), symptomatic/known CSE (n = 28), and other CSE (n = 12). Subjects were followed up at a mean 8.5 (1.0SD, range: 6.3–10.2) years post-CSE. Full-scale intelligence quotient data were available for 70 subjects with CSE and GMS for 65.

### 3.2 Sociodemographic, clinical characteristics

Full-scale intelligence quotient and GMS of participants according to CSE, CSE etiological subcategory, and HC group are presented in Supporting Information 4. Sociodemographic and clinical characteristics, FSIQ, and GMS of participants according to groups with CSE +, CSE −, and HC are provided in Table 1. A greater proportion of both children with CSE + and CSE − had major MRI abnormalities compared with controls. A greater proportion of those with CSE − were right handed, and a greater proportion of those with CSE + had previous CSE at initial enrollment into the inception cohort compared with children with CSE −. Children with CSE + vs CSE − were older at the time of follow-up in the current study, but the follow-up interval for both groups was the same. There were no other intergroup differences.

**Table 1:** Sociodemographic and clinical characteristics of participants according to HC, subjects with CSE +, and subjects with CSE −.

	HC (n = 72)	CSE + (n = 22)	CSE − (n = 48)
Sex^c^			
Male	29 (60%)	12 (55%)	22 (46%)
Female	43 (40%)	10 (45%)	26 (54%)
Age (years)^d^ mean ± SD			
At CSE^d^		3.8 ± 2.5	2.8 ± 3.0
At follow-up^d^	12.1 ± 3.0	13.0 ± 3.1	11.2 ± 3.4
Time until follow-up (years)^d^ mean ± SD		8.8 ± 1.0	8.4 ± 0.9
IMD^d^ mean ± SD		36 ± 14	32 ± 14
Handedness^c^			
Right	55 (76%)	15 (68%)^a^	40 (83%)^b^
Left	9 (13%)	6 (27%)^a^	3 (6%)^b^
Unknown	8 (11%)	1 (5%)	5 (11%)
Seizures^c^			
First ever (incident)		17 (77%)^a^	46 (96%)^b^
Recurrent		5 (23%)^a^	2 (4%)^b^
Febrile		11 (50%)	34 (71%)
Focal		10 (46%)	14 (29%)
Seizure character^c^			
Intermittent		11 (50%)	25 (52%)
Continuous		11 (50%)	23 (48%)
Seizure type^c^			
Focal		10 (46%)	14 (29%)
Generalized		12 (54%)	34 (71%)
Duration (mins)^d^ mean ± SD		74.6 ± 31.7	77.9 ± 76.5
Major MRI abnormalities^c^	0 (0%)^a^, ^b^	7 (32%)	7 (15%)
Preterm birth^c^		5 (23%)	5 (10%)

Abbreviations: HC = healthy controls, CSE = convulsive status epilepticus, CSE + = subjects with lower cognitive ability, CSE − = subjects without lower cognitive ability, PFS = prolonged febrile seizures, SD = standard deviation, IMD = Index of Multiple Deprivation.

a p ≤ 0.05 compared with subjects with CSE −.

b p ≤ 0.05 compared with subjects with CSE +.

c Fisher's exact test.

d Kruskall–Wallis H test.

### 3.3 Subcortical nuclei volumes across HC group, groups with CSE + and CSE −

**Fig. 3:**
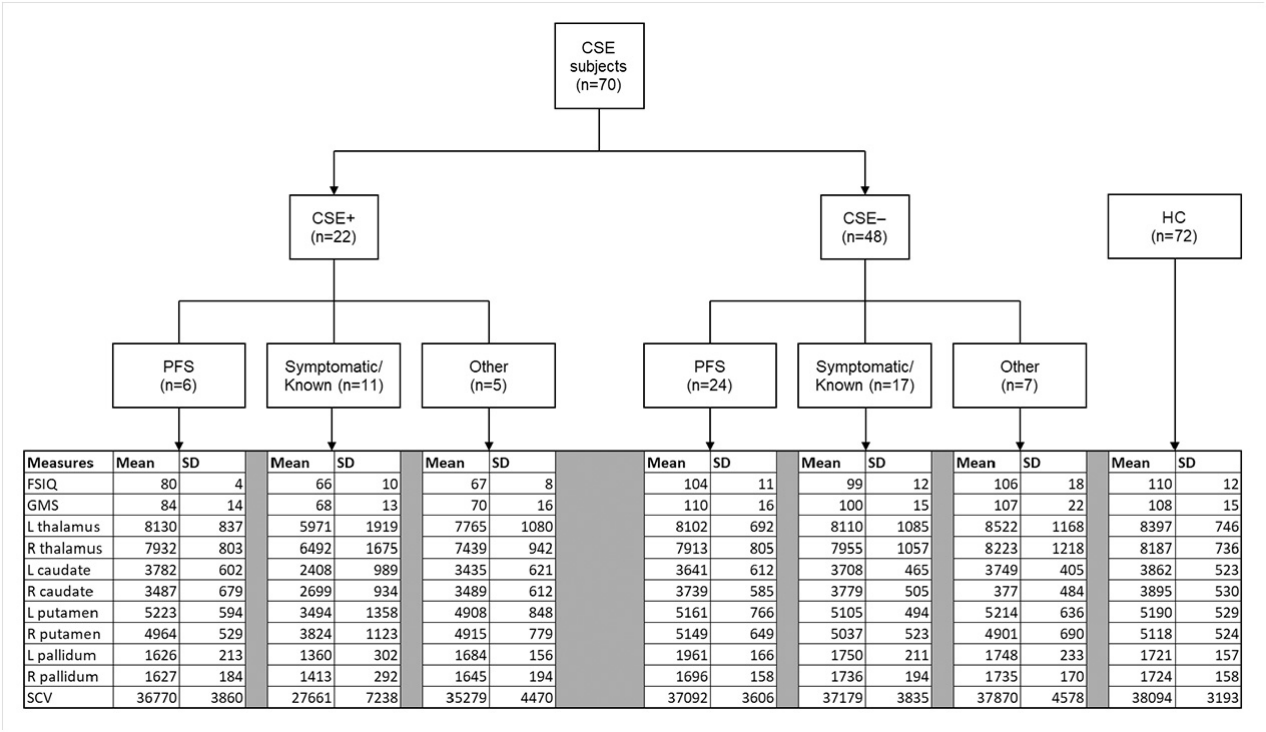
Stratification of subjects with CSE into cognitive groups: CSE + (FSIQ < 85) and CSE − (FSIQ > 85) with etiological subgroups and key cognitive and volumetric data. CSE = convulsive status epilepticus; HC = healthy controls; FSIQ = full-scale IQ; IMD = Index of Multiple Deprivation; ICV = intracranial volume; GM = gray matter; ANCOVA = univariate analysis of covariance; GMS = Global Memory Score; L = left; R = right; SCV = total subcortical volume.

Of the 70 CSE cases, 22 were CSE + (PFS (n = 6), symptomatic/known (n = 11), other (n = 5)) and 48 were CSE − (PFS (n = 24), symptomatic/known (n = 17), other (n = 7)). Key cognitive and volumetric data are presented in Fig. 3. No significant differences were observed for any subcortical volumes between the HC groups and the group with CSE − (Table 2).

**Table 2:** Subcortical volumes comparing healthy controls (HC), CSE cases with lower cognitive ability (CSE +), and CSE cases without lower cognitive ability (CSE −).

Subcortical structures	Mean structure volume (mm^3^) (standard deviation)			Brain volumes adjusted for in ANCOVA model	Mean volume difference CSE + vs HC (95%CI) (mm^3^)	Mean volume difference CSE − vs HC (95%CI) (mm^3^)	Mean volume difference CSE + vs CSE − (95%CI) (mm^3^)
	HC (n = 72)	CSE + (n = 22)	CSE − (n = 48)				
Left thalamus	8397 (746)	6968 (1789)	8166 (912)	ICV	**− 633 (− 1007, − 259)** **p** **=** **0.001**⁎	− 88 (− 270, − 95) p = 0.345	− 548 (− 1010, − 86) p = 0.021
				GM	**− 560 (− 912, − 209)** **p** **=** **0.002**⁎	0 (− 198, 196) p = 0.995	− 582 (− 1019, − 144) p = 0.010
				Cortical/cerebellar	**− 618 (− 981, − 254)** **p** **=** **0.001**⁎	− 6 (− 209, 198) p = 0.956	**− 644 (− 1100, − 189)** **p** **=** **0.006**
Right thalamus	8187 (736)	7100 (1441)	7973 (947)	ICV	− 358 (− 661, − 55) p = 0.021	− 64 (− 252, 124) p = 0.500	− 305 (− 693, 81) p = 0.120
				GM	− 355 (− 677, − 33) p = 0.031	20 (− 190, 230) p = 0.854	− 380 (− 783, 23) p = 0.064
				Cortical/cerebellar	− 402 (− 732, − 71) p = 0.018	14 (− 202, 231) p = 0.897	− 433 (− 850, − 17) p = 0.042
Left caudate nucleus	3862 (523)	3016 (1014)	3680 (529)	ICV	**− 421 (− 711, − 131)** **p** **=** **0.005**⁎	− 114 (− 295, 67) p = 0.213	− 323 (− 671, 25) p = 0.068
				GM	− 402 (− 693, − 111) p = 0.007	− 87 (− 275, 100) p = 0.359	− 340 (− 681, 1) p = 0.051
				Cortical/cerebellar	**− 434 (− 729, − 139)** **p** **=** **0.004**⁎	− 92 (− 280, 98) p = 0.340	− 373 (− 720, − 26) p = 0.036
Right caudate nucleus	3895 (530)	3093 (871)	3759 (533)	ICV	**− 389 (− 647, − 132)** **p** **=** **0.003**⁎	− 76 (− 251, 100) p = 0.394	− 370 (− 688, − 52) p = 0.023
				GM	**− 415 (− 692, − 139)** **p** **=** **0.004**⁎	− 39 (− 221, 143) p = 0.674	− 430 (− 761, − 98) p = 0.012
				Cortical/cerebellar	**− 444 (− 724, − 165)** **p** **=** **0.002**⁎	− 43 (− 227, 141) p = 0.644	− 459 (− 795, − 123) p = 0.008
Left putamen	5190 (529)	4287 (1331)	5149 (651)	ICV	− 425 (− 753, − 96) p = 0.012	25 (− 152, 202) p = 0.782	− 498 (− 940, − 56) p = 0.028
				GM	− 381 (− 702, − 61) p = 0.020	73 (− 106, 253) p = 0.419	− 496 (− 918, − 74) p = 0.022
				Cortical/cerebellar	− 419 (− 744, − 93) p = 0.012	− 69 (− 114, 251) p = 0.456	− 536 (− 965, − 107) p = 0.015
Right putamen	5118 (524)	4383 (1054)	5073 (607)	ICV	− 302 (− 562, − 42) p = 0.023	26 (− 124, 177) p = 0.728	− 311 (− 618, − 3) p = 0.048
				GM	− 282 (− 543, − 21) p = 0.034	76 (− 80, 232) p = 0.334	− 342 (− 646, − 39) p = 0.028
				Cortical/cerebellar	− 315 (− 580, − 49) p = 0.021	73 (− 86, 232) p = 0.364	− 376 (− 686, − 66) p = 0.018
Left pallidum	1721 (157)	1506 (286)	1720 (191)	ICV	− 91 (− 160, − 21) p = 0.011	32 (− 13, 76) p = 0.165	− 117 (− 203, − 31) p = 0.009
				GM	− 95 (− 169, 20) p = 0.013	43 (− 7, 93) p = 0.089	**− 137 (− 230, 44)** **p** **=** **0.004**⁎
				Cortical/cerebellar	− 104 (− 180, − 28) p = 0.008	42 (− 9, 93) p = 0.103	**− 147 (− 242, − 52)** **p** **=** **0.003**⁎
Right pallidum	1724 (158)	1524 (262)	1716 (171)	ICV	− 72 (− 132, 12) p = 0.019	22 (− 16, 60) p = 0.256	**− 114 (− 188, − 39)** **p** **=** **0.003**⁎
				GM	− 79 (− 147, − 11) p = 0.022	35 (− 8, 78) p = 0.114	**− 136 (− 219, 53)** **p** **=** **0.002**⁎
				Cortical/cerebellar	− 88 (− 158, − 19) p = 0.013	34 (− 11, 78) p = 0.134	**− 145 (− 230, − 60)** **p** **=** **0.001**⁎
Total subcortical (SCV)	38,094 (3193)	31,876 (7156)	37,236 (3757)	ICV	**− 2690 (− 4019, − 1361)** **p** **<** **0.001**⁎⁎	–-237 (− 1010, 536) p = 0.545	**− 2585 (− 4392, − 778)** **p** **<** **0.006**⁎⁎
				GM	**− 2570 (− 3924, − 1216)** **p** **=** **0.001**⁎⁎	121 (− 735, 977) p = 0.780	**− 2844 (− 4650, − 1037)** **p** **=** **0.003**⁎⁎
				Cortical/cerebellar	**− 2824 (− 4244, − 1403)** **p** **<** **0.001**⁎⁎	92 (− 799, 983) p = 0.838	**− 3113 (− 5006, − 1221)** **p** **=** **0.002**⁎⁎

Volumes have been adjusted for sex, age at follow-up, and one of the following: ICV, GM, or cortical/cerebellar volume.

Bold = statistically significant results (p < 0.05).

⁎ Significantly different volumes after Bonferroni correction, p < 0.05.

⁎⁎ Significantly different volumes, p < 0.05.

Adjusting for age at follow-up, sex, and ICV, significantly lower volumes were observed in the group with CSE + compared with HC for the following structures: left thalamus (p = 0.001), left caudate nucleus (p = 0.005), and right caudate (p = 0.003). Significant differences were still observed when ICV was substituted with GM or cortical/cerebellar volumes, except for the left caudate nucleus, the difference of which became nonsignificant on substitution with GM.

When volumes between subjects with CSE + and CSE − were compared, only differences in the right pallidum were statistically significant for all 3 models incorporating ICV, GM, or cortical/cerebellar volume. Left pallidum volumes were significantly lower in the group with CSE + in models that adjusted for GM and cortical/cerebellar volumes (p = 0.004, p = 0.003). The left thalamus volume was significantly lower in the group with CSE + in only one model (cortical/cerebellar, p = 0.006).

Total subcortical volume was significantly lower in the group with CSE + compared with both HC (p < 0.001) and the group with CSE − (p = 0.002–0.006) in all three ANCOVA models.

### 3.4 Association between subcortical nuclei volumes and cognition

Two of the HCs had LCA (FSIQ = 82, FSIQ = 83), and in linear regression models for HCs including age, sex, and ICV, SCV was not significantly associated with any measures of cognition. Comparatively, 31% of subjects with CSE presented with LCA at follow-up. Regression models including controls vs subjects with CSE as a variable (adjusting for age at follow-up, sex, ICV, and subcortical volumes) demonstrated that history of CSE was a significant predictor of FSIQ scores (β = − 0.339, p < 0.001). Total subcortical volume remained a significant predictor in these models for both cognitive outcomes: FSIQ (β = 0.384, p = 0.001) and GMS (β = 0.439, p = 0.001).

In regression analyses restricted to subjects with CSE, with adjustment for potential confounders (age at follow-up, sex, IMD, duration, ICV, and etiology), several individual subcortical nuclei volumes were associated with FSIQ and GMS scores, but after Bonferroni correction, these regression coefficients were non-significant (Table 3). However, SCV emerged as an independent predictor of FSIQ (β = 0.492, p = 0.005) and GMS (β = 0.453, p = 0.014) (Table 3). As expected, given their collinearity, this model was very similar if SCV was substituted by ICV or symptomatic vs non-symptomatic/known etiology (Supporting Information 4). When stepwise regression approach was used, SCV remained as the significant association ahead of ICV and etiology (Supporting Information 4).

**Table 3:** Association between subcortical nuclei volumes and cognitive measures expressed as standardized β coefficients.

Subcortical volumes	FSIQ		GMS	
	β	p value	β	p value
Left thalamus	0.443	0.009	0.451	0.015
Right thalamus	0.317	0.074	0.347	0.073
Left caudate nucleus	0.282	0.031	0.268	0.044
Right caudate nucleus	0.243	0.071	0.335	0.019
Left putamen	0.316	0.017	0.251	0.082
Right putamen	0.236	0.126	0.120	0.481
Left pallidum	0.405	0.011	0.344	0.053
Right pallidum	0.350	0.037	0.412	0.024
Total (SCV)	0.492	0.005⁎⁎	0.453	0.014⁎

Linear regression of neurocognitive measures using subcortical nuclei volume as a predictor, correcting for sex, age at follow-up, Index of Multiple Deprivation, intracranial volume, duration of CSE, and etiology. For individual subcortical volumes, p values are not significant after Bonferroni correction. Abbreviations: FSIQ = full-scale intelligence quotient, GMS = Global Memory Score, β = standardized beta coefficient.

⁎ p ≤ 0.05.

⁎⁎ p ≤ 0.005.

Unless specified, no other covariates, including socioeconomic status were significantly associated with the dependent variable.

## 4 Discussion

### 4.1 Summary of findings

In this novel follow-up study of a population-based CSE cohort, we find that 8.5 years post-CSE, (1) subcortical volumes were independently reduced in subjects with CSE + compared with HCs and subjects with CSE − and (2) a possible role for measuring subcortical nuclei as a biomarker of cognition in childhood CSE, although measuring ICV or classifying children with CSE according to etiology would be similarly predictive of FSIQ and or GMS.

A number of subcortical structures were found to be significantly lower in subjects with CSE + compared with HC, even after adjusting for GM and cortical/cerebellar volumes. Comparatively, there were no significant differences in subcortical volumes between HC and subjects with CSE −, and as FSIQ and GMS scores are similar between these groups, it is possible that this is due to preservation of subcortical volumes.

### 4.2 Significance of findings

Since it has been reported that individual subcortical volumes are reduced across numerous etiologies of epilepsy [33] and that volumes are associated with cognition independently of the cortex in early onset childhood [11], it would have been reasonable to consider that a similar relationship may exist in childhood CSE. However, this was not observed in the current study. Instead, we find that there are lower volumes of the left thalamus and bilateral caudate nuclei in the group with CSE + compared with HC, and while the left thalamic volume is reduced in the group with CSE + compared with that in the group with CSE −, it is the bilateral pallidi volumes, rather than caudate nuclei, that are lower. Therefore, all structures excluding the putamina are implicated in some form in the group with CSE +. Using SCV rather than individual volumes in linear regression analyses was found to be significantly associated with LCA. One potential explanation of this is that our study was underpowered to detect difference related to individual volumes. However, another possibility, given the heterogeneity of individual subcortical structures reported to be associated with LCA, is that the SCV or subcortical networks are implicated.

The total subcortical nuclei volume was found to be associated with both measures of cognition in subjects with CSE, even after adjusting for CSE etiology in linear regression models. However, no such relationship was observed in controls alone. Together, these data suggest that there are factors specific to subjects with CSE that influence the association between subcortical volume and cognition.

To the authors' knowledge, only one group has specifically studied subcortical volumes in the context of status epilepticus and this was an adult-based study [34]. This retrospective study found 17 of the 225 patients undergoing MRI for investigation of seizures to have pulvinar MRI abnormalities; all 17 presented with status epilepticus. While this would suggest that these subcortical volumetric differences are frequent in status epilepticus, the majority of these patients were found to have a preexisting lesion secondary to trauma or stroke, so it is uncertain if the findings were due to the etiology or the subcortical volume. In our study, we had a similar challenge, since the subjects with symptomatic/known etiology had smaller subcortical volumes and were the group that had the worst cognitive scores. When we examined the subjects with CSE only, subcortical volume or ICV or etiology were the main factors associated with cognition scores but only when used singly rather than considered together in the analyses, reflecting the marked collinearity.

It is possible that subcortical nuclei contribute to cognitive deficits post-CSE in a more complex manner than simply subcortical volumetric differences alone. The role of the subcortical structures has typically been overlooked in favor of a predominantly corticocentric approach to understanding cognition [35]. This is thought to be primarily due to historical biases, but with the advent of neuroimaging modalities throughout the 20th century, our understanding of the importance of reciprocal corticostriatal pathways in cognition has evolved. Considering the association between SCV and cognition, we speculate that there may be a disruption of subcortical networks resulting in such deficits. It has been proposed that the basal ganglia provide an inhibitory control of generalized absence seizures [36]. If this control is exerted across all epilepsies, defects in subcortical networks may be indicative of a predisposition to seizures. Tractography of diffusion MRI data in the current cohort as well as others could help elucidate the contribution of white matter connections to cognition in CSE and perhaps seizure control.

In vivo status epilepticus models have demonstrated that it is not only neuronal death that occurs post-CSE but also neural remodeling [37], which may disrupt inhibitory networks between subcortical nuclei. In a study of the subjects with PFS of the STEPSOUT cohort, tract-based spatial statistics has demonstrated white matter microstructural reorganization [21]. None of these subjects had LCA; however, this reorganization perhaps represents a compensatory change that occurs secondary to reduced structural volumes. It is possible that these microstructural changes may be associated with LCA in other etiological groupings, such as subjects with symptomatic/known CSE as observed in our study.

### 4.3 Study limitations

While our study has the advantage of prospective, long-term follow-up of a large population-based cohort, it has limitations. Loss to follow-up is inevitable in long-term cohort studies, and dropouts reduced our cohort to 31% of the size of NLSTEPSS [38]. A lower proportion of subjects with symptomatic/known CSE comprised the follow-up cohort in the current study compared with inception cohort. As subcortical volumetric differences among subjects with CSE +, HC, and CSE − are largely driven by this etiology, our findings of lower subcortical volume post-CSE are perhaps an underestimate. We acknowledge that the power of some of our etiology subgroups is relatively small. To mitigate this, we focused on the differences between subjects with/without LCA while adjusting for etiology.

Neuropsychological assessments and neuroimaging were not systematically carried out at the occurrence of the initial CSE of cohort members, although developmental history was obtained. Thus, it is not possible to be certain if the observations in the current study are the direct consequence of the initial CSE event, whether they are preexisting or if these changes have resulted by other means. We carried out multiple comparisons, which increased our chances of Type 1 errors. However, to mitigate against, this we applied a Bonferroni correction.

We accept that there may be questions about our definition of LCA being FSIQ < 85, but this represents a threshold of less than 1 SD below the instrument mean. Other studies have used the same definition to detect subtle/mild LCA rather than intellectual disability defined as FSIQ < 70 (2 SDs below the instrument mean) [[39], [40], [41]].

Identifying, prioritizing, and supporting vulnerable patients in their relatively premorbid states could allow for improvements in quality of life and adult-life opportunities. Cognitive rehabilitation therapies may provide support in susceptible children, particularly computer-based interventions allowing for greater accessibility and thereby satisfactory compliance [42]. Factors associated with neurocognitive deficits in patients with CSE, such as SCV, could in future be implemented into scoring systems that would guide the allocation of psychological interventions. Volumetric analysis is not routinely performed in neuroradiological assessments. However, FSL, the software used in this study, could feasibly be automated for the use in clinical practice to compare patient volumes against a control-based reference range.

## 5 Conclusion

After long-term follow-up post-childhood CSE, subcortical nuclei volumes are significantly lower in children who have LCA compared with those that do not. However, in this cohort, we are unable to determine whether the relationship is independent of ICV or etiology. Future, larger scale studies may help tease this out.

## Funding sources

Funding: STEPSOUT was funded by the 10.13039/501100000691Academy of Medical Sciences, the 10.13039/501100000355BUPA Foundation (BUPA 22094612), the 10.13039/100010269Wellcome Trust (SGCL1Chin), the National Institute for Health Research Biomedical Research Centre, University College London at Great Ormond Street Hospital for Children NHS Foundation Trust and Young Epilepsy. RCS is supported by the Great Ormond Street Hospital Children's Charity. MY is supported by NHS Research Scotland and the 10.13039/501100000848University of Edinburgh. KHB and RFMC are supported by the 10.13039/501100000848University of Edinburgh.

## Declaration of competing interest

None of the authors have any competing interests to disclose.

## Appendix A Supplementary data

Supplementary tables Image 1

**Supplemental Information:** Our STROBE Statement is available online. Supplementary data to this article can be found online at https://doi.org/10.1016/j.yebeh.2020.107119.
