# Supplementary material for: Subcortical nuclei volumes are associated with cognition in children post-convulsive status epilepticus: Results at nine years follow-up
Source: Epilepsy Behav. 2020 Sep;110:107119. doi: 10.1016/j.yebeh.2020.107119 (PMC7479509; doi:10.1016/j.yebeh.2020.107119)
Supplement: Supplementary file 1 — Supplementary tables [file mmc1.docx]

SUPPORTING INFORMATION 1: Pearson correlation coefficients between dependent variables, covariates and independent variables.

|  | | | | | | | | | | | | | | | | | |  |
| --- | --- | --- | --- | --- | --- | --- | --- | --- | --- | --- | --- | --- | --- | --- | --- | --- | --- | --- |
| Pearson Correlation | FSIQ | Sex | Age at follow-up | IMD | Duration of CSE | PFS | Symptomatic/Known | Other | ICV | Left thalamus volume | Right thalamus volume | Left caudate nucleus volume | Right caudate nucleus volume | Left putamen volume | Right putamen volume | Left globus pallidus volume | Right globus pallidus volume | SCV |
| FSIQ | 1.000 | -0.058 | -0.243* | -0.108 | -0.104 | 0.320** | -0.279* | -0.058 | 0.447** | 0.561** | 0.501** | 0.512** | 0.481** | 0.533** | 0.492** | 0.518** | 0.468** | 0.583** |
| Sex | -0.058 | 1.000 | -0.033 | -0.049 | 0.097 | -0.033 | -0.035 | 0.089 | 0.454** | 0.236* | 0.261* | 0.040 | 0.072 | 0.182 | 0.255* | 0.256* | 0.304* | 0.220* |
| Age at follow-up | -0.243* | -0.033 | 1.000 | 0.029 | -0.052 | -0.478** | 0.305* | 0.231* | 0.012 | -0.052 | 0.013 | -0.150 | -0.099 | -0.092 | -0.080 | 0.023 | 0.064 | -0.068 |
| IMD | -0.108 | -0.049 | 0.029 | 1.000 | 0.292* | 0.043 | -0.134 | 0.118 | -0.070 | 0.007 | -0.007 | 0.114 | 0.051 | -0.094 | -0.005 | 0.028 | 0.062 | 0.009 |
| Duration of CSE | -0.104 | 0.097 | -0.052 | 0.292* | 1.000 | 0.078 | -0.145 | 0.086 | -0.097 | -0.025 | -0.083 | 0.055 | 0.108 | -0.053 | -0.039 | -0.040 | 0.035 | -0.018 |
| PFS | 0.320** | -0.033 | -0.478** | 0.043 | 0.078 | 1.000 | -0.707** | -0.394** | 0.154 | 0.204* | 0.160 | 0.222* | 0.168 | 0.259* | 0.268* | 0.090 | 0.105 | 0.229* |
| Symptomatic/Known | -0.279* | -0.035 | 0.305* | -0.134 | -0.145 | -0.707** | 1.000 | -0.371** | -0.221* | -0.314** | -0.221* | -0.291* | -0.222* | -0.336** | -0.293* | -0.190 | -0.174 | -0.310* |
| Other | -0.058 | 0.089 | 0.231* | 0.118 | 0.086 | -0.394** | -0.371** | 1.000 | 0.085 | 0.140 | 0.076 | 0.087 | 0.068 | 0.096 | 0.028 | 0.128 | 0.088 | 0.101 |
| ICV | 0.447** | 0.454** | 0.012 | -0.070 | -0.097 | 0.154 | -0.221* | 0.085 | 1.000 | 0.786** | 0.813** | 0.543** | 0.585** | 0.611** | 0.749** | 0.770** | 0.785** | 0.797** |
| Left thalamus volume | 0.561** | 0.236* | -0.052 | 0.007 | -0.025 | 0.204* | -0.314** | 0.140 | 0.786** | 01.000 | 0.918** | 0.742** | 0.698** | 0.794 | 0.760** | 0.849** | 0.816** |  |
| Right thalamus volume | 0.501** | 0.261* | 0.013 | -0.007 | -0.083 | 0.160 | -0.221* | 0.076 | 0.813** | 0.918** | 1.000 | 0.633** | 0.722** | 0.629 | 0.794** | 0.793** | 0.871** |  |
| Left caudate nucleus volume | 0.512** | 0.040 | -0.150 | 0.114 | 0.055 | 0.222* | -0.291* | 0.087 | 0.543** | 0.742** | 0.633** | 1.000 | 0.800** | 0.757 | 0.634** | 0.709** | 0.663** |  |
| Right caudate nucleus volume | 0.481** | 0.072 | -0.099 | 0.051 | 0.108 | 0.168 | -0.222* | 0.068 | 0.585** | 0.698** | 0.722** | 0.800** | 1.000 | 0.598 | 0.699** | 0.707** | 0.775** |  |
| Left putamen volume | 0.533** | 0.182 | -0.092 | -0.094 | -0.053 | 0.259* | -0.336** | 0.096 | 0.611** | 0.794** | 0.629** | 0.757** | 0.598** | 1.000 | 0.695** | 0.784** | 0.652** |  |
| Right putamen volume | 0.492** | 0.255* | -0.080 | -0.005 | -0.039 | 0.268* | -0.293* | 0.028 | 0.749** | 0.760** | 0.794** | 0.634** | 0.699** | 0.695** | 1.000 | 0.736** | 0.853** |  |
| Left globus pallidus volume | 0.518** | 0.256* | 0.023 | 0.028 | -0.040 | 0.090 | -0.190 | 0.128 | 0.770** | 0.849** | 0.793** | 0.709** | 0.707** | 0.784** | 0.736** | 1.000 | 0.844** |  |
| Right globus pallidus volume | 0.468** | 0.304* | 0.064 | 0.062 | 0.035 | 0.105 | -0.174 | 0.088 | 0.785** | 0.816** | 0.871** | 0.663** | 0.775** | 0.652** | 0.853** | 0.844** | 1.000 | - |
| SCV | 0.583** | 0.220* | -0.068 | 0.009 | -0.018 | 0.229* | -0.310* | 0.101 | 0.797** |  |  |  |  |  |  |  |  |  |

Abbreviations: FSIQ = Full-scale IQ, IMD = Index of Multiple Deprivation, PFS = Prolonged Febrile Seizures, ICV = intracranial volume. SCV = Total subcortical volume. *p<0.05, **p<0.005

SUPPORTING INFORMATION 2: Demographic and clinical features of excluded subjects.

|  | Excluded (n=6) |
| --- | --- |
| Sex |  |
| Male | 4 |
| Female | 2 |
| Age (years)  mean ± SD |  |
| At CSE | 4.3 ± 1.9 |
| At follow-up | 13.2 ± 2.1 |
| Time until follow-up (years)  mean ± SD | 9.0 ± 0.6 |
| IMD  mean ± SD | 29.3 ± 11.7 |
| Handedness |  |
| Right | 4 |
| Left | 2 |
| Seizures |  |
| First ever (incident) | 4 |
| Recurrent | 2 |
| Febrile | 2 |
| Focal | 5 |
| Seizure character |  |
| Intermittent | 3 |
| Continuous | 3 |
| Seizure onset |  |
| Focal | 0 |
| Focal to bilateral tonic–clonic seizure | 5 |
| Primary generalised | 1 |
| Duration (mins)  mean ± SD | 75.8 ± 26.3 |
| Major MRI abnormalities | 6 |
| Preterm | 2 |

All excluded participants belonged to the Symptomatic CSE aetiology. Abbreviations: SD = standard deviation, CSE = convulsive status epilepticus, IMD = Index of Multiple Deprivation

SUPPORTING INFORMATION 3: MRI reports, aetiology and cognitive and memory scores for excluded subjects.

| MRI report | Aetiology | FSIQ | GMS |
| --- | --- | --- | --- |
| Right middle cerebral artery infarct | Symptomatic/Known | 57 | - |
| Previous germinal matrix haemorrhage, right frontoparietal porencephalic cyst, left side normal | Symptomatic/Known | 78 | - |
| Hypoxic ischaemic encephalopathy, right hemi-atrophy, left cerebellar diaschisis, periventricular signal change on left | Symptomatic/Known | 52 | - |
| Germinal matrix haemorrhage, mainly on left with left hippocampal sclerosis, some white matter damage on right | Symptomatic/Known | 66 | - |
| Compensated obstructive hydrocephalus with white matter loss | Symptomatic/Known | 76 | 50 |
| Compensated hydrocephalus, white matter loss, scarring in right dorsolateral midbrain | Symptomatic/Known | - | - |

Abbreviations: FSIQ = Full-scale IQ, GMS = Global Memory Score.

SUPPORTING INFORMATION 4: Standardised beta coefficients and p values for exploratory linear regression analyses.

| Linear regression adjustment | | FSIQ | | | | | | GMS | | | | | |
| --- | --- | --- | --- | --- | --- | --- | --- | --- | --- | --- | --- | --- | --- |
|  |  | SCV | | ICV | | Symptomatic vs non-symptomatic | | SCV | | ICV | | Symptomatic vs non-symptomatic | |
|  |  | Beta | p | Beta | p | Beta | p | Beta | p | Beta | p | Beta | p |
| All | | 0.489 | 0.005 | 0.143 | 0.440 | -0.064 | 0.551 | 0.459 | 0.012 | 0.104 | 0.606 | -0.101 | 0.398 |
| SCV | | 0.613 | <0.001 |  |  |  |  | 0.557 | <0.001 |  |  |  |  |
| ICV | |  |  | 0.600 | <0.001 |  |  |  |  | 0.529 | <0.001 |  |  |
| Symptomatic vs non-symptomatic | |  |  |  |  | -0.260 | 0.039 |  |  |  |  | -0.250 | 0.058 |
| Stepwise regression | 1 | 0.583 | <0.001 |  |  |  |  | 0.467 | <0.001 |  |  |  |  |
|  | 2 | 0.569 | <0.001 |  |  |  |  |  |  |  |  |  |  |
|  | 3 | 0.613 | <0.001 |  |  |  |  |  |  |  |  |  |  |

Linear regression analyses of FSIQ and GMS adjusting for: SCV, ICV and Symptomatic/Known vs Non-Symptomatic/Known; SCV; ICV; Symptomatic/Known vs Non-Symptomatic/Known; SCV, ICV and Symptomatic/Known vs Non-Symptomatic/Known in a step-wise regression. Adjustment is made for sex, age at follow-up, index of multiple deprivation, and duration of CSE. Abbreviations: FSIQ = Full-scale intelligence quotient, GMS = Global Memory Scale, SCV = total subcortical volume, ICV = intracranial volume.
